# Copper and zinc concentrations in uterine fluid and blood serum during the estrous cycle and pre-pubertal phase in water buffaloes

**Published:** 2015-09-15

**Authors:** Sayed Mortaza Alavi Shoushtari, Siamak Asri Rezaie, Amir Khaki, Abulfazl Belbasi, Hamid Tahmasebian

**Affiliations:** 1 *Department of Theriogenology, Faculty of Veterinary Medicine, Urmia University, Urmia, Iran; *; 2 *Department of Internal Medicine and Clinical Pathology, Faculty of Veterinary Medicine, Urmia University, Urmia, Iran; *; 3 *Graduate Student, Faculty of Veterinary Medicine, Urmia University, Urmia, Iran.*

**Keywords:** Buffalo, Estrous cycle, Uterine fluid, Copper, Zinc

## Abstract

To investigate uterine fluid and serum copper (Cu) and zinc (Zn) variations during the estrous cycle in water buffaloes, 71 genital tracts and blood samples were collected from the abattoir in Urmia, Iran. The phase of the estrous cycle was determined by examining ovarian structures; 18, 15, 16 and 22 were pro-estrous, estrous, met-estrous and diestrous, respectively. The uterine fluid was collected by gentle scraping of the uterine mucosa with a curette. Blood serum and uterine fluid samples of 71 pre-pubertal buffalo calves were also collected and treated in similar manners. The mean (± SEM) total serum (77.10 ± 1.50 µg dL^-1^) and uterine fluid (296.40 ± 9.40 μg dL^-1^) Cu in cyclic cows was higher than the values of 54.00 ± 1.10 μg dL^-1^ and 133.40 ± 5.70 μg dL^-1^ in pre-pubertal calves, respectively. Blood serum (114.60 ± 3.20 μg dL^-1^) and the uterine fluid (349.90 ± 8.90 μg dL^-1^) Zn content in cyclic cows were also higher than those (98.80 ± 1.50 μg dL^-1^ and 246.6 ± 4.50 μg dL^-1^ respectively) in pre-pubertal calves. Serum Cu in pro-estrus and estrus were lower than those in other stages and also lower than those in the uterine fluid. The lowest serum Zn content was recorded in pro- and met-estrus, while in the uterine fluid it was observed in estrus. In all stages of estrous cycle the uterine fluid Zn content was significantly higher than those of the serum. These results suggested that during the estrous cycle in the buffalo cows, Cu and Zn were actively secreted in uterine lumen and were not dependent on blood serum. The values also increased after puberty.

## Introduction

The uterus by creating a suitable environment for gamete survival and transport and early embryonic development has an important role in the reproduction of domestic animals. To do this task, the uterus should secrete a variety of substances. 

Copper (Cu) is necessary for many enzymes like the Cu–Zn–superoxide-dismutase (SOD), which is involved in cell protection against free (oxygen) radicals. Copper is also needed for the cytochrome C oxidase that is responsible for energy supply and for cellular and humoral immunity.^1^ Elevated copper concentrations reduce oxidative processes and glucose consumption that may cause immotility and reduced viability of the spermatozoa.^1^^,^^2^ Copper is involved in hypophyseal receptors function and controls the release of LH from pituitary gland.^3^ Copper also affects female reproductive performance and its deficiency may result in structural and biochemical abnormalities in the fetus.^3^ A review of symptoms of copper deficiency^4^ and the functions of copper in the dairy cows are available.^5^^,^^6^

Zinc (Zn) is closely related to the cell biochemistry, physiology and morphology. It is involved in enzyme functions and protein and carbohydrate metabolism, and its concentration in the uterine secretion functions as an intra- and extracellular cation regulatory mechanism.^7^ Zinc plays a role as an activator in several enzyme systems and involves in cell replication and differentiation, particularly in nucleic acid metabolism.^8^

Ions including Cu and Zn, move through the epithelial cells of the uterus into the lumen of the reproductive tract causing a concentration gradient which in turn helps in an osmotic gradient providing the driving force to transport water by osmosis out of the epithelial cells into the uterine lumen. Leese pointed out that ion concentration and their movement are essential for the regulation of enzyme activity and of the pH of the uterine fluid.^9^ The ionic composition of uterine fluid is apparently derived from combination of ions from the blood and ions secreted from uterine epithelium.^10^

Despite the importance of Cu and Zn concentrations in the uterine fluid and its role in gamete, zygote and early embryo development, there is little published information available on the Cu and Zn contents of the uterine fluid during the estrous cycle in buffaloes.

This study was carried out to investigate changes in Cu and Zn concentrations in the uterine fluid during the estrous cycle of the buffalo cow, to compare them with concentrations in the blood serum during the estrous cycle to find any possible relationship between them and to compare their total values with those in pre-pubertal calves to investigate possible variations in their level during the course of puberty. This information may be used in preparing culture media for *in vitro* fertilization (IVF) and other assisted reproductive technologies in buffaloes.

## Materials and Methods

Genital tract and blood sample of 232 slaughter buffalo cows were collected in Urmia abattoir (37˚ 33΄ N, 45˚ 4΄ E) from November 2011 to June 2012. Body condition scores (BCS; score 2.5 to 4) and ages of the animals (2 to 4 years) were recorded before sample collection. Samples of animals with BCS of less than 2.5 and more than 4 (On the scale of: 1 = emaciated to 5 = obese) were not collected. Blood samples were collected by jugular vein puncture in plain test tubes before slaughter and left to clot. Samples were quickly transferred to the lab in a cold box. In an initial examination, immature, pregnant and abnormal genital samples or samples with hemolyzed blood samples were discarded. Genital tracts were examined to determine the stage of their cycles by examining the structures (fully developed follicles and corpora lutea, regressed corpora lutea and graafian follicles) on their ovaries as described by Noakes.^11^ Out of 71 normal cyclic genital tracts selected for further examination, 18 cases were pro-estrous, 15 estrous, 16 met-estrous and 22 diestrous. Seventy one pre-pubertal calf genital tracts (with growing follicles but without corpus luteum) were also selected. Serum samples were harvested by centrifuging the clotted blood at 3000 rpm for 10 min, and stored in microtubes (Eppendorf, Hamburg, Germany) at – 20 ˚C until examination. 

Uterine fluid samples were collected by gentle scraping of the mucosa by a curette after incisions made in both uterine horns and stored in the microtubes at –20 ˚C until examination. Samples with any abnormal endometrial appearance or discharge were also discarded; a smear prepared from suspected uterine samples was tested for the presence of poly-morphonuclear leucocytes after standard Giemsa staining. Eventually, a total number of 71 serum and uterine fluid samples were used in the study. The uterine fluid and blood serum Cu and Zn contents of the samples were determined by spectrophotometry (Model M330 spectrophotometer; Camspec, Cambridge, UK) using commercial kits (EliTech Diagnostic, Sees, France) for Cu and Zn after thawing the samples at room temperature. The uterine fluid samples were diluted (1:10) before estimation.

The data was analyzed by using SPSS software (Version 18; SPSS Inc., Chicago, USA) computer program. Data was analyzed by one way ANOVA, statistic mean and standard error of mean was calculated for each group, serum and uterine fluid samples in the groups were compared by paired student’s *t*-test and limit for statistical significance was set at *p* ≤ 0.05. 

## Results

The mean ± SEM of total Cu concentrations recorded in serum and uterine fluid samples in cyclic samples were significantly (*p *< 0.05) higher than those in pre-pubertal samples (Table 1). Mean serum and uterine fluid total Zn concentrations in cyclic samples showed a significant (*p* ≤ 0.01) difference with those in pre-pubertal samples (Table 1).

**Table 1:** Total copper and zinc concentrations (Mean ± SEM) in serum and uterine fluid of cyclic and pre-pubertal buffalo samples

**Parameters**	**No.**	**Cyclic**	**Pre-pubertal**
**Serum** **Cu (** **μg dL** ^-1^ **)**	71	77.10 ± 1.50*	54.00 ± 1.10
**Zn (** **μg dL** ^-1^ **)**	71	114.60 ± 3.20*	98.80 ± 1.50
**Uterine fluid** **Cu (** **μg dL** ^-1^ **)**	71	269.40 ± 9.40*	133.40 ± 5.70
**Zn (** **μg dL** ^-1^ **)**	71	349.90 ± 8.90*	246.60 ± 4.50

* indicates significant difference between columns at *p* < 0.01.

The mean ± SEM of Cu concentrations of serum samples in pro-estrus and estrus were not significantly different, but in met-estrus and diestrus it was different and the highest value was recorded in diestrus. The mean serum Cu values Uterine fluid Cu content in pro-estrus was the highest and that in diestrus was the lowest value (Table 2). Uterine fluid Cu values in all stages of the estrous cycle were significantly (*p* < 0.01) higher than those of the serum samples (Table 2).

**Table 2:** Copper concentration (Mean ± SEM) in serum and uterine fluid in different phases of estrus cycle of the buffalo

**Phases **	**No.**	**Serum (** **μg dL** ^-1^ **)**	**Uterine fluid (** **μg dL** ^-1^ **)**
**Pro-estrus**	18	63.00 ± 1.50^a^*	395.40 ± 6.50^a^
**Estrus**	15	63.20 ± 1.90^a^*	240.50 ± 7.30^b^
**Met-estrus**	16	81.60 ± 1.70^b^*	228.20 ± 8.00^bc^
**Diestrus**	22	89.00 ± 2.10^c^*	215.90 ± 4.60^c^

* indicates significant difference between columns at *p* < 0.01.

abc different superscripts indicate significant difference in each column at *p* < 0.05.

The mean (± SEM) serum Zn concentrations obtained in estrus and diestrus were significantly higher than that in pro-estus and met-estrus. The mean Zn values of the uterine fluid samples in pro-estrus, estrus, met-estrus and diestrus were all different (*p* < 0.05), (Table 3). 

The uterine fluid Zn concentrations in different phases of the estrous cycle showed a significant difference (*p* < 0.01) with those in the serum samples (Table 3).

**Table 3:** Zinc concentration (Mean ± SEM) in serum and uterine fluid in different phases of estrus cycle of the buffalo

**Phases **	**No.**	**Serum (** **μg dL** ^-1^ **)**	**Uterine fluid (** **μg dL** ^-1^ **)**
**Pro-estrus**	18	92.40 ± 0.60^a^*	320.6 ± 2.80^a^
**Estrus**	15	111.40 ± 2.70^b^*	238.8 ± 2.90^b^
**Met-estrus**	16	93.20 ± 0.50^a^*	367.6 ± 4.90^c^
**Diestrus**	22	150.60 ± 3.60^c^*	436.9 ± 4.70^d^

* indicates significant difference between columns at *p* < 0.01.

abc different superscripts indicate significant difference in each column at *p* < 0.05.

## Discussion

Little published information is available concerning the concentration of Cu and Zn in the blood serum and uterine fluid or their variations during the different phases of the estrous cycle in buffaloes. This work was an attempt to get some information in this field. 

Collecting the uterine secretions in buffaloes, as it is in the bovine, bears some problems. Flushing the uterus through the cervical canal and flushing the exposed uterus through a laparotomy section has been reported as techniques of collecting uterine discharges. The former has the problem of concentrating the flushing, and side effects of a surgical operation are the sequelae of the latter. In addition, these techniques could be carried out only in experimental animals and sometimes impractical. The procedure of collecting uterine samples in this study has none of these problems. Age of the animals was recorded by examining their teeth and their plain of nutrition was determined by recording their body condition score before slaughter, which have been reported to influence the serum Cu and Zn contents.^12^^,^^13^ The age of animals was considered to be in a range of 3 to 6 years at the time of selecting samples. For pre-pubertal samples, presence of small growing follicles and the absence of any form of corpus luteum were considered as the animal being at pre-pubertal state. The exact dietary composition of the buffalo cows ration before slaughter was not available but, by estimating the body condition score (BCS) of the animals at the time of slaughter, we sampled only those animals that had BCS of more than 2.5 (in a 1 to 5 scale) and less than 4, which meant that, at least, they have not been under- or overfed. In this way, samples from animals in the same age and body condition group were collected to minimize the effect of age and plain of nutrition on serum and uterine fluid Cu and Zn contents. In addition, serum total Cu and Zn concentrations obtained here were compared with standard values reported in the literature for the bovine.

Blood and Radostits and Radostits *et al*. have reported that the mean copper in blood plasma of cattle is 1.26 ± 0.31 mg mL^-1^ (126.00 ± 31.00 μg dL^-1^) which may vary according to the state of reproduction, plain of nutrition and age of the animal, and consider values between 57.00 to 19.00 μg dL^-1^ as lower normal range.^12^^,^^13^ These values are much lower than serum total copper content of 77.10 ± 1.50 μg dL^-1^ obtained in this study, although we emphasized on the buffalo state of the reproduction only, and also lower than the total values we previously obtained for the bovine (66.10 ± 6.50 μg dL^-1^). The same authors have reported that the zinc value in cattle serum is between 80.00 to120.00 μg dL^-1^. Total serum zinc content of 114.60 ± 3.20 μg dL^-1^ recorded in this study for buffaloes is in agreement with the values of their report. Also, our results show that the sampled animals have not been Zn deficient before slaughter. Baxter reported the value of 9.00 to 26.00 µmol L^-1^ (57.00 to 164.00 μg dL^-1^) for the serum of normal cattle, and regarded the figure of 9.00 µmol L^-1^ (57.00 μg dL^-1^) as the minimum normal level.^14^ He has reported the plasma zinc content of normal cattle as 9.00 to 18.00 µmol L^-1^ (41.00 to 82.00 μg dL^-1^), and considers the values less than 9.00 μmol L^-1^ (41.00 μg dL^-1^) as abnormal. Our results in buffalo (114.60 ± 3.20 μg dL^-1^) were much higher than these values, as were total zinc values we reported previously for the bovine (91.90 ± 5.40 μg dL^-1^).^15^

 Akhtar *et al*. in comparison of plasma Cu and Zn concentrations of anestrous buffalo cows with those of cyclic cows found that these parameters in anestrous cows are significantly less than those in cyclic cows.^16^ They concluded that Cu and Zn deficiencies, by themselves or in combination, may be responsible for the anestrous state in the animals, and Cu and Zn supplementation can improve their situation. Copper and zinc supplementation of beef cows improves their pregnancy rates after AI, and plasma Cu and Zn concentrations in supplemented cows are higher than those in controls.^17^ Furthermore, this supplementation reduces the incidence of uterine infections, embryonic loss and endometrial scar formation, leading to a better post-partum uterine involution and fertility.^14^ However, Griffiths *et al*. found no difference in mean open days and days to services in Cu and Zn supplemented dairy cows and controls.^18^

Verdugo *et al*. studied *in vitro* effects of Cu and Zn on the rat uterine musculature and found that these two cations have contradictory effect; Zn^++^ has suppressive effect while Cu^++^ is stimulatory.^19^ Ho *et al*. found that homozygote rats with genetic defects of Cu-Zn superoxide dismutase had a lower fertility rates than heterozygous normal rats, which was considered as a result of more embryonic loss.^20^ El-Hendy *et al*. observed that in rats fed with Zn deficient diets the weight gain is less than those in controls and have lower hematological parameters and serum Cu and Zn concentrations.^21^

Impairment of the reproductive performance in the female and spermatogenesis in the male may be the symptoms of animal zinc deficiency,^22^ and zinc by exerting an antioxidant activity and kreatinizing the teat canal mucosa has an effect in preventing mastitis in the first week of lactation in dairy cows.^23^ Furthermore, zinc has an effect on postpartum fertility of the dairy cow, and exerts its action by reducing the effects of stress induced by udder infections and foot problems and by stimulating immunity system function.^22^

In this study, it was found that total Cu and Zn content of both serum and uterine fluid in cyclic animals were higher than those in pre-pubertal animals. It seems that hormonal and other changes that take place during the course of puberty in buffaloes have an increasing effect on the body Cu and Zn concentrations including blood serum and uterine fluid. 

In this study mean uterine fluid Cu concentrations in all the stages of the estrous cycle in buffaloes were much more than those in the serum, while in the bovine, it was only higher in pro-estrus and diestus (187.20 ± 47.70 μg dL^-1^ and 355.90 ± 88.90 μg dL^-1^, respectively).^15^ This may be the effect of an active secretion of Cu from endometrial cells into the uterine lumen. This secretion was more prominent in follicular phase (pro-estrus and estrus) of the cycle than that in the luteal phase in buffaloes. This can be explained by the elevated circulatory estradiol concentration and increased blood flow to the uterus in this phase. Blood serum Cu content, however, was higher in met-estrus and diestrus (luteal phase) suggesting that in buffaloes, as in the bovine, progesterone may possibly have a role in increasing the serum, but not the uterine fluid, Cu concentrations. Buffalo uterine fluid Cu content in met-estrus was lower than pro-estrus and estrus and the lowest level recorded in diestrus. This reveals a contradictory effect of progesterone on the serum and uterine fluid Cu content.

Michaluk and Kochman reported that combination of Cu and gonadotropin-releasing hormone (GnRH) in releasing follicle stimulating hormone (FSH) and luteinizing hormone (LH) from the anterior pituitary is more effective than that of the natural GnRH.^3^ This may be an explanation for the lower fertility in Cu deficient animals, however, is contrary to our results, because in pro-estrus and estrus, in which the peak of FSH and LH occur, serum Cu content was at a low level. It is possible that the surge of these gonadotropins has a decreasing effect on the regulators of Cu concentrations in the serum but not in the uterus. It is also possible that the transmitters that cause gonadotropin surge release directly or via elevation of estradiol, which follows the gonadotropin surge, affect Cu pool in the body to decrease serum, but not the uterine fluid Cu content in these phases of the cycle.

In this study uterine fluid Zn concentrations in all the phases of the cycle were more than those in the serum. The differences were all highly significant. The buffalo uterine fluid Zn content in estrus, as it was in the bovine (216.90 ± 61.30 μg dL^-1^), was the lowest. This is the period in which the uterus is under the dominance or effects of estrogen dominance, which suggests that estradiol by affecting secretory functions of the uterine mucosa dilutes the Zn concentration in the uterine fluid. Blood serum zinc content, on the other hand, was at the highest level in diestrus and at a high level in estrus that was contrary to the uterine level in the latter phase, while, in the bovine the lowest serum Zn content (78.70 ± 9.10 μg dL^-1^) was observed in met-estrus.^15^

It is suggested that these data to be confirmed by further investigations carried out on a bigger population of animals and in controlled conditions in association with a detailed examination of hormonal changes.

It could be concluded that the cyclical changes in buffalo serum and uterine tissues leads to the elevation of copper and zinc concentrations in the uterine fluid and the amount of this elevation varies according to the stage of the estrous cycle. The course of events at puberty has also an increasing effect on their serum and uterine fluid concentrations. Giving an insight into the physiology of uterus during the estrous cycle and pre-pubertal phase, this information may be useful in culture media preparation for IVF and other assisted reproductive technologies in the buffalo.
